# Decreased telomere length in the small airway epithelium suggests accelerated aging in the lungs of persons living with human immunodeficiency virus (HIV)

**DOI:** 10.1186/s12931-018-0821-0

**Published:** 2018-06-13

**Authors:** Stella Xu, Emily A. Vucic, Tawimas Shaipanich, Stephen Lam, Wan Lam, Julio S. Montaner, Don D. Sin, S. F. Paul Man, Janice M. Leung

**Affiliations:** 1 Centre for Heart Lung Innovation, University of British Columbia, Vancouver, Canada; 2 British Columbia Cancer Agency, Vancouver, Canada; 3 Division of Respiratory Medicine, University of British Columbia, Vancouver, Canada; 4 BC Centre for Excellence in HIV/AIDS, University of British Columbia, Vancouver, Canada

**Keywords:** HIV, Telomere length, Epithelial cell

## Abstract

Human immunodeficiency virus (HIV) infection is associated with an increased risk of chronic obstructive pulmonary disease (COPD) independent of cigarette smoke exposure. Previous studies have demonstrated that decreased peripheral leukocyte telomere length is associated with HIV, suggesting an accelerated aging phenomenon. We demonstrate that this process of telomere shortening also occurs in the lungs, with significant decreases in telomere length observed in small airway epithelial cells collected during bronchoscopy. Molecular evidence of accelerated aging in the small airway epithelium of persons living with HIV may be one clue into the predisposition for chronic lung disease observed in this population.

## To the editor

Persons living with human immunodeficiency virus (PLWH) face a growing burden of age-related conditions including chronic obstructive pulmonary disease (COPD) [1]. One proposed etiology for this phenomenon is the concept of “inflamm-aging” [2] whereby PLWH, even those on chronic antiretroviral therapy, continue to have low-grade inflammation driving cellular turnover. An important biomarker of this particular form of accelerated biologic aging is telomere length, a measure of replicative senescence. Telomeres, made up of repeated TTAGGG sequences, are the ends of chromosomes that provide structural integrity during the process of cell division. With each division cycle, the fidelity of replicating these ends is imperfect with the loss of approximately 25 to 200 base pairs (bp) with each division [3]. Ultimately, a critical telomere length is reached whereby cell proliferation ceases and apoptosis ensues. We have previously demonstrated that peripheral leukocyte telomere lengths in PLWH are significantly shorter than in HIV-negative control subjects, with the severity of lung function as measured by forced expiratory volume in 1 second (FEV1) correlating with decreased telomere length [4]. In this study, we further these findings by determining whether advanced replicative senescence can also be observed within the lungs of PLWH.

PLWH were drawn from the patient population at St. Paul’s Hospital in Vancouver, Canada, a tertiary care setting with an active bronchoscopy program and HIV outpatient clinic. Eligible PLWH were patients with documented HIV-1 infection who were undergoing bronchoscopies for clinical purposes (i.e. for lung masses or nodules or to rule out infection) with additional research specimen collection added on to the procedure. All subjects were ≥ 19 years old and provided written informed consent under the University of British Columbia (UBC) Providence Health Care ethics protocol H14–03267. HIV-negative control subjects were recruited from participants of a lung cancer screening study at the British Columbia Cancer Agency in Vancouver, Canada. With the exception of three PLWH who were lost to follow up, subjects underwent spirometry according to American Thoracic Society/European Respiratory Society guidelines using the Crapo reference equations [5]. 24 PLWH and 41 HIV-negative participants were enrolled.

Small airway epithelial (SAE) cells were obtained in regions of the lung that were free from lung nodules, masses, or infiltrates, as determined by chest computed tomography scans. Cytologic brushings were taken via flexible bronchoscopy, with brushings occurring in the order of the 5th–6th generation airways where resistance to the brush was met. Airway epithelial cell telomere length was measured by quantitative polymerase chain reaction methods originally outlined by O’Callahan and Fenech [6]. Standard curves were generated from known quantities of synthesized oligomers of telomere (*TEL*) DNA [(TTAGGG)_14_] and single copy gene (*36B4*) DNA [CAGCAAGTGGGAAGGTGTAATCCGTCTCCACAGACAAGGCCAGGACTCGTTTGTACCCGTTGATGATAGAATGGG]. Sample telomere DNA length was then measured based on the ratio of telomere DNA length to *36B4 (RPLP0)* DNA length as obtained from their respective curves.

Differences in demographic characteristics between PLWH and the HIV-negative cohort were assessed using t-tests or Mann-Whitney U tests for continuous variables (depending on the normality of distribution) and Fisher’s exact tests for categorical variables. The relationships between telomere length and continuous variables were determined using Pearson correlation tests. T-tests were used to evaluate telomere length differences between PLWH and HIV-negative controls in a univariate model. Given differences in demographics between the two cohorts with respect to age, sex distribution, and smoking pack-years, a linear regression model adjusting for these factors was performed to determine whether the differences between PLWH and HIV-negative telomere lengths persisted. Because the PLWH cohort was predominantly male, a secondary analysis was performed using only male participants.

Demographic characteristics of the cohort are listed in Table 1. PLWH were significantly younger with a greater proportion of males. Lung function as measured by FEV1/forced vital capacity (FVC) and FEV1 %Predicted was similar between the two groups as were the proportion of current, former, and never smokers. HIV-negative patients had significantly greater smoking pack-years. 71% of PLWH had an undetectable viral load with a mean CD4 count of 462 cells/mm^3^.

**Table 1:** Characteristics of the study cohort

Characteristic	PLWH (*n* = 24)	HIV- (*n* = 41)	*p*-value
Age	58.13 ± 9.14	62.59 ± 6.70	0.044
Male sex (%)	20 (83%)	23 (56%)	0.031
FEV1 (% Predicted)	76.79 ± 15.84	76.24 ± 24.02	0.579
FEV1/FVC (%)	68.57 ± 12.70	66.39 ± 15.43	0.697
Smoking status			
Current	12 (50%)	19 (46%)	0.184
Former	10 (42%)	22 (54%)	
Never	2 (8%)	0	
Smoking Pack-Years	29.54 ± 31.09	48.98 ± 13.53	< 0.001
Undetectable Plasma HIV Viral Load	17 (71%)	–	–
CD4 Count (cells/mm^3^)	462 ± 306	–	–
Telomere Length (kbp/genome)	108.13 ± 45.51	171.53 ± 52.20	< 0.001

Despite the fact that PLWH were on average 4 years younger than HIV-negative patients, their telomere lengths were significantly shorter (108.13 ± 45.51 kilobase pair (kbp)/genome for PLWH, 171.53 ± 52.20 kbp/genome for HIV-negative patients, *p* < 0.001). This relationship remained significant (*p* < 0.001) after adjustment for sex, smoking pack-years, and age. Given the differences in sex distribution between the two groups and the previously reported differences in telomere lengths between males and females, a second analysis was performed with just male participants (mean age 59.96 years for male PLWH and 60.73 years for HIV-negative males, *p* = 0.724). Male PLWH still had significantly shorter telomere lengths compared to HIV-negative males (104.45 ± 44.67 kbp/genome vs. 178.51 ± 52.84 kbp/genome, *p* < 0.001). After adjustment for age and smoking pack-years, the relationship between HIV serostatus and shorter telomere length in only males remained significant (*p* < 0.001).

The relationships between telomere length and other variables of interest are shown in Tables 2 and 3. Consistent with a previous study in which smoking appears to attenuate the relationship between age and telomere length in SAE cells, telomere length in our predominantly smoking cohort was not significantly correlated with age [7]. Telomere length was also not significantly correlated with FEV1/FVC, FEV1 %Predicted, COPD status (as defined by a FEV1/FVC < 70%), smoking pack-years, or sex. Within PLWH, telomere length was not different between those with detectable and undetectable viral loads. There was also no correlation between telomere length and CD4 count.

**Table 2:** Telomere length associations with continuous variables

Variable	R	*p*-value
Age	0.089	0.479
Smoking Pack-Years	0.118	0.353
FEV1/FVC	−0.040	0.760
FEV1% Predicted	−0.068	0.604
CD4 cell count (PLWH only)	−0.015	0.946

**Table 3:** Telomere length associations with dichotomous variables

Variable	Telomere length mean ± SD (kbp/genome)	*p*-value
Sex		
Male	144.07 ± 61.33	0.414
Female	156.06 ± 52.27	
Viral Load (PLWH Only)		
Detectable	104.77 ± 70.74	0.561
Undetectable	116.29 ± 48.10	
COPD (all patients)		
Yes	158.311 ± 56.09	0.510
No	148.45 ± 59.01	
COPD (PLWH only)		
Yes	104.94 ± 43.08	0.518
No	119.62 ± 52.17	
COPD (HIV-negative only)		
Yes	174.56 ± 49.59	0.686
No	167.66 ± 56.58	

In this study, we provide the first molecular evidence for accelerated aging within the SAE of PLWH, many of whom had abnormal lung function. The difference in telomere length was nearly double what was found between healthy male smokers and non-smokers in a previous study looking at SAE telomere lengths [7]. Importantly, the majority of PLWH in this study had sufficient immune recovery and undetectable plasma HIV viral loads, and our findings were significant even after accounting for cigarette smoke exposure. Two possible mechanisms might explain our observations: first that HIV accelerates epithelial cell turnover in the small airway or second that HIV impacts the telomere lengths of basal stem/progenitor cells that give rise to the SAE. Either of these possibilities may be the result of the virus itself. The effect of HIV on the SAE has recently been investigated in two studies. One suggests that the presence of X4-tropic virus increases epithelial cell permeability and the expression of pro-inflammatory cytokines [8], while another has found that HIV interacts with the basal epithelial layer to create a tissue-destructive phenotype driven by matrix metalloproteinases [9]. Whether HIV can replicate within the epithelial cell is a matter of contention [8, 10], regardless, one effect on the SAE layer may be the promotion of a particularly injurious and inflammatory environment by which senescence mechanisms are triggered. As shortened telomere lengths are a measure of replicative senescence, our work supports this hypothesis. Future work into functionally characterizing the senescent nature of these cells and the impact of their presence on surrounding lung parenchymal, stromal, and immune cells is ongoing and of great interest.

The question of whether or not replicative senescence in the SAE contributes to the development of future COPD within PLWH requires larger prospective and longitudinal studies. Unlike a previous study from our group in which peripheral leukocyte telomere length was associated with lung function in PLWH, we did not find decrements in lung function to be significantly associated with SAE telomere shortening. We suspect that our small sample size may have left us underpowered to detect such a signal or that the distribution of lung function in our cohort skewed towards more severe disease. Repeat measurements in a larger cohort with a wide distribution of lung function should be performed. Investigation of telomere length in asymptomatic PLWH without apparent lung disease and in HIV cohorts with a greater proportion of females will also be critical in extending these findings to larger populations. Moreover, other measurements of aging such as methylation changes, senescence-associated beta-galactosidase, and DNA damage markers may provide further evidence of a global aging process in HIV. In summary, we describe here an important aging phenotype within a critical cell layer of the small airway that may distinguish PLWH from their uninfected counterparts.

## Abbreviations

COPD: Chronic obstructive pulmonary dsiease
FEV1: Forced expiratory volume in 1 second
FVC: Forced vital capacity
HIV: Human immunodeficiency virus
PLWH: Persons living with HIV
SAE: Small airway epithelium
TL: Telomere length
